# Correlation between olfactory function, age, sex, and cognitive reserve index in the Italian population

**DOI:** 10.1007/s00405-022-07311-z

**Published:** 2022-02-24

**Authors:** Carla Masala, Annachiara Cavazzana, Fabrizio Sanna, Maria Paola Cecchini, Alice Zanini, Flavia Gasperi, Leonardo Menghi, Isabella Endrizzi, Monica Borgogno, Serena Drago, Elena Cantone, Andrea Ciofalo, Alberto Macchi, Giulia Monti, Valentina Parma, Maria Piochi, Ilenia Pinna, Luisa Torri, Giorgia Cabrino, Giancarlo Ottaviano, Alfonso Luca Pendolino, Angela Pignatelli, Faride Pighin, Vincenzo Bochicchio, Gaetano Motta, Giorgia Fontana, Benedetta Pasquariello, Carlo Cavaliere, Valentina Iacono, Thomas Hummel

**Affiliations:** 1 Department of Biomedical Sciences, University of Cagliari, SP8 Cittadella Universitaria Monserrato, 09042 Cagliari, Italy; 2 Department of Otorhinolaryngology, Smell and Taste Clinic, TU Dresden, Dresden, Germany; 3 Section of Anatomy and Histology, Department of Neurosciences, Biomedicine and Movement Sciences, University of Verona, Strada Le Grazie, 8, 37134 Verona, Italy; 4 Research and Innovation Centre, Fondazione Edmund Mach, Via E. Mach, 1, San Michele All’Adige, 38010 Trento, Italy; 5 Mérieux NutriSciences Italia, Prato, Italy; 6 Neurosciences, Reproductive and Odontostomatologic Sciences, Unit of Ear, Nose and Throat, Federico II University, Naples, Italy; 7 ORL Clinica, University of Insubria and Varese, ASST Settelaghi, Varese, Italy; 8 Monell Chemical Senses Center, 3500 Market Street, Philadelphia, 19104 USA; 9 University of Gastronomic Sciences, Pollenzo, Cuneo, Italy; 10 Department of Neuroscience, ENT Section, University of Padua, Padua, Italy; 11 Department of Neuroscience and Rehabilitation, University of Ferrara, Ferrara, Italy; 12 Department of Humanistic Studies, University of Calabria, Rende, Italy; 13 Clinic of Otorhinolaryngology, Head and Neck Surgery Unit, Department of Anesthesiology, Surgical and Emergency Science, University of Campania “Luigi Vanvitelli”, Naples, Italy; 14 Geriatric Unit A, Azienda Ospedaliera Universitaria Integrata, Verona, Italy; 15 Unit of Rhinology, Department of Organi di Senso, University La Sapienza, Rome, Italy; 16 Division of Nephrology and Dialysis, “Santa Maria della Misericordia” Hospital, Rovigo, Italy; 17 Center Agriculture Food Environment, University of Trento/Fondazione Edmund Mach, San Michele all’Adige, Trento, Italy; 18 Department of Technology and Innovation, University of Southern Denmark, Odense, Denmark

**Keywords:** Olfactory function, Smell, Sniffin’ Sticks, Olfaction, Identification, Chemosensory system

## Abstract

**Purpose:**

Loss of smell decreases the quality of life and contributes to the failure in recognizing hazardous substances. Given the relevance of olfaction in daily life, it is important to recognize an undiagnosed olfactory dysfunction to prevent these possible complications. Up to now, the prevalence of smell disorders in Italy is unknown due to a lack of epidemiological studies. Hence, the primary aim of this study was to evaluate the prevalence of olfactory dysfunction in a sample of Italian adults.

**Methods:**

Six hundred and thirty-three participants (347 woman and 286 men; mean age 44.9 years, SD 17.3, age range 18–86) were recruited from 10 distinct Italian regions. Participants were recruited using a convenience sapling and were divided into six different age groups: 18–29 years (*N* = 157), 30–39 years (*N* = 129), 40–49 years (*N* = 99), 50–59 years (*N* = 106), > 60 years (*N* = 142). Olfactory function, cognitive abilities, cognitive reserve, and depression were assessed, respectively, with: Sniffin’ Sticks 16-item Odor Identification Test, Montreal Cognitive Assessment, Cognitive Reserve Index, and the Beck Depression Inventory. Additionally, socio-demographic data, medical history, and health-related lifestyle information were collected.

**Results:**

About 27% of participants showed an odor identification score < 12 indicating hyposmia. Multiple regression analysis revealed that OI was significantly correlated with age, sex, and cognitive reserve index, and young women with high cognitive reserve index showing the highest olfactory scores.

**Conclusion:**

This study provides data on the prevalence of olfactory dysfunction in different Italian regions.

## Introduction

Olfactory function plays a key role in human life [1] regulating food ingestion, emotional responses, social and reproductive behavior [2]. Olfactory function decreases over the lifespan [3–5] and approximately 5% of the general population exhibits functional anosmia [6]. People with olfactory disorders showed increased risk in food poisoning and cooking, or heating gas injuries due to their inability to identify spoiled food or to detect a gas leak [2, 7, 8]. For instance, participants with olfactory deficits showed impairments in food intake, social life, cognitive function, and personal hygiene with a negative impact in daily life [2, 9–11]. Loss of olfactory function is closely linked to both mood and affective disorders in younger and older adults [12]. On average, olfactory function in humans changes not only in relation to age [3–5], but also in relation to sex [13–16], cultural differences in olfactory experience [17, 18], genetic factors [19], infections [20], head trauma [12], and neurodegenerative diseases [21–24], or emotional disorders [25].

Olfactory function in humans is often evaluated by odor threshold which partly reflects the anatomy of the nasal cavity [26], the expression of olfactory receptors in the nasal epithelium [27] and the olfactory bulb volume [28, 29]. In contrast, odor identification and discrimination are more strongly associated with cultural differences and involve cognitive functions to a relatively larger degree [30]. In addition, a positive correlation was found between cultural and typical food odors, pleasantness, and identification of a smell [31]. Hence, because of these experience-dependent factors, odor identification tests have been adapted to different countries in order to account for these cultural/regional differences [32–37].

In Italy, to our knowledge, only few studies investigated olfactory function in healthy subjects. In particular, Eibenstein and colleagues in 2005 [38], through the Sniffin’ Sticks Screening test (Identification test with 12 items), assessed the familiarity and identification of the 12 odors in Italian normosmic subjects, while the Maremmani’s study [39] assessed the validity and reliability of the Italian Olfactory Identification Test (IOIT) [38, 39]. Later, Cantone and colleagues evaluated regional differences in the odor hedonic perception of an odor, but only in the cities of Padua, Rome, Naples and Siracusa, using the Sniffin’ Sticks Identification test (16 items) [40].

Still, up to now, the exact prevalence of olfactory dysfunction in Italy remains unknown, and particularly its relation to different variables of interest, such as age, sex, cognitive ability, cognitive reserve, and level of depression has not fully explored.

Considering these findings, we aimed to perform a more comprehensive investigation of olfactory function on a wider sample of Italian subjects, focusing on the prevalence of olfactory dysfunction in different Italian regions representative of the North, Centre and South of Italy. In addition, we related these data with age, sex, cognitive ability, cognitive reserve index, and depression level.

## Methods

### Participants

**Fig. 1:**
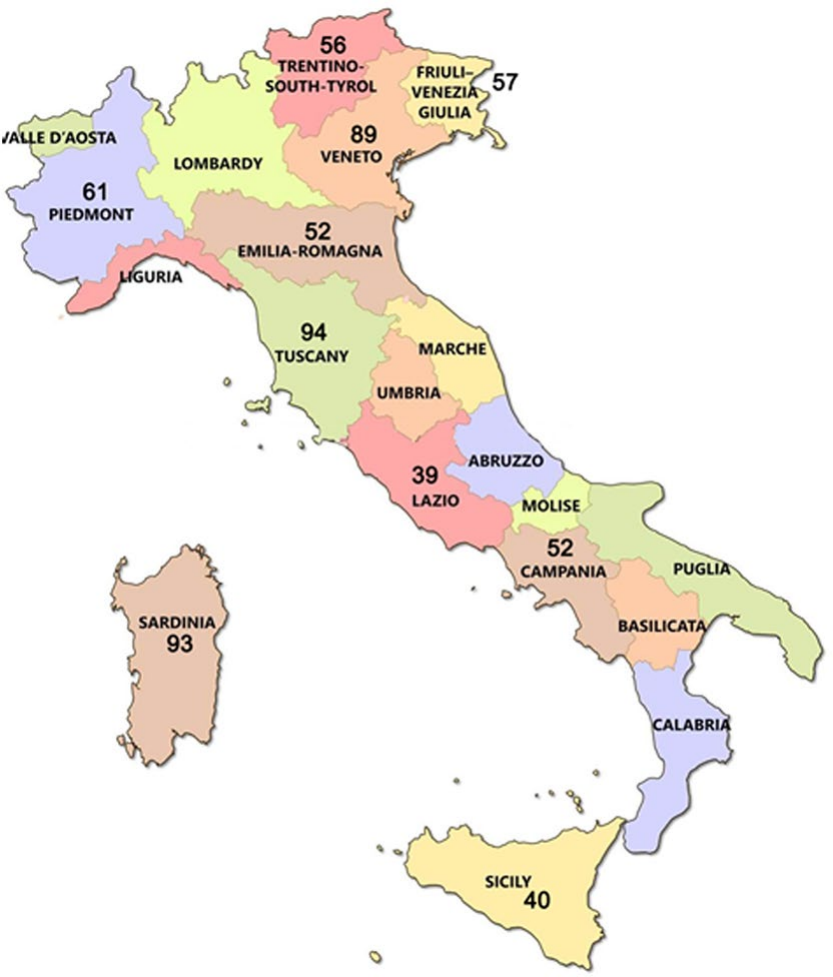
Sample distribution in the Italian regions involved in the study

A total of 633 participants (347 women and 286 men) were recruited from 10 different Italian regions (mean age 44.9 years, SD 17.3, age range 18–86, Fig. 1), namely: Sardinia (*N* = 93), Trentino (*N* = 56), Lazio (*N* = 39), Campania (*N* = 52), Tuscany (*N* = 94), Piedmont (*N* = 61), Friuli-Venezia Giulia (*N* = 57), Sicily (*N* = 40), Emilia-Romagna (*N* = 52), Veneto (*N* = 89). Participants were recruited using a convenience sapling and were divided into six different age groups: 18–29 years (*N* = 157), 30–39 years (*N* = 129), 40–49 years (*N* = 99), 50–59 years (*N* = 106), > 60 (*N* = 142). The study was conducted simultaneously in the 10 Italian regions from September 2018 to December 2019 in pre-COVID-19 pandemic. The exclusion criteria were the following: history of neurologic disease (such as epilepsy, brain tumor, Parkinson's disease, Alzheimer disease), major head injury, local respiratory tract factors such as active rhinitis or sinusitis (allergic or infectious) at the moment of the testing, and any cancer or treatment for cancer (chemotherapy or head or neck radiation).

The demographic/clinical interview for each participant included age, sex, employment, weight (kg), height (cm), body mass index (BMI, calculated as ratio of height and weight, expressed as kg/m^2^), current medications, and smoking history.

### Ethical standard

This study was performed in accordance with the Declaration of Helsinki on Biomedical Studies for human subjects. Informed written consent was obtained from all participants. The study design was approved by the Ethics Committee of the University Hospital of Cagliari (Prot. Number: NP/2018/1630).

### Procedures

Olfactory function, depression, cognitive reserve, and cognitive function were evaluated, respectively, as follows: “Sniffin’ Sticks” 16-item odor Identification (OI) test (Burghart, Wedel, Germany) [41–43], Beck Depression Inventory (BDI), Cognitive Reserve Index (CRI) [44], Montreal Cognitive Assessment (MoCA) [45]. All questionnaires and examinations were performed for each participant in one session in a well-ventilated room with little or no background odor. Exclusion criteria were age < 18 years, otolaryngology disorders, severe renal dysfunction, neurodegenerative disorders or other comorbidities influencing olfactory function, as well as dementia and psychiatric conditions interfering with the study participation.

Olfactory function was assessed with a standardized and reliable OI (Burghart, Wedel, Germany) which consists of 16 common odors presented together with four verbal descriptors in a multiple forced-choice format (three distractors and one target). Updated normative data reported a score in OI test was ≥ 12 correct answers [43].

The cognitive status was screened through the Italian version of the Montreal Cognitive Assessment (MoCA), which measures cognitive abilities in different domains: attention and concentration, executive functions, memory, language, visual-constructional skills, conceptual thinking, calculations, and spatial orientation [45]. The total possible score of the MoCA test was 30 and any score ≥ 26 was considered normal.

In addition, the cognitive reserve was quantified by using the “Cognitive Reserve Index (CRI)” [44]. This self-reported questionnaire quantifies the amount of cognitive reserve acquired during a person’s lifetime. CRI conveys three main sources: education, working activity, and leisure time activities. Each of these features (items) of an individual’s lifetime is recorded as a sub-score.

The depression was evaluated by means of the Beck Depression Inventory II (BDI II) [46], which is a self-reported questionnaire with 21 items examining how participants have been feeling during the last two weeks. Each item was rated in increasing severity from 0 to 3. The overall depression level was classified as minimal (= 0–13), mild (= 14–19), moderate (= 20–28), and severe depression (= 29–63) according to the sum of the item ratings. The total experimental procedure required approximately 75–90 min.

### Statistical analysis

A sample size calculation was performed to assess the required minimum number of subjects to be enrolled in the study. Based on previous studies using similar protocols [5, 21, 47, 48], a minimum number of about 450 total subjects was considered adequate to detect investigated differences. In fact, a power calculation, considering a critical effect size *f* = 0.20–0.25 (medium effect), with 95% power and a 5% significance level in a standard two-way ANOVA, suggested a minimal required number of about 450 total subjects, and a power calculation considering a critical effect size *f*^2^ = 0.10–0.15 (medium effect), with 95% power, and a 5% significance level for each investigated factor in a multiple linear regression model, suggested a required minimum number of about 200 total subjects.

Data are presented as mean values ± standard deviation (SD). At first, between-subject one-way ANOVA with sex, age, BDI, MoCA, and CRI as covariates was performed to assess differences in the OI due to the region of origin for each participant. The effect of age and gender on OI, MoCA, CRI, and BDI was assessed by separate two-way ANOVAs. For significant effects from ANOVAs, multiple pairwise comparisons were performed with the Tukey’s (HSD) test in the case of significant interactions, or with Bonferroni’s corrected pairwise *t* tests in all the other cases.

In addition, effect size estimations (Cohen’s *d* for any significant pairwise comparison) were also reported in the Results section where appropriate (a value of 0.2, 0.5 or 0.8 indicates a small, medium or large effect size, respectively).

To identify the more promising factors for the multivariate regression analyses, bivariate correlations between the OI versus sex, age, MoCA, CRI, and BDI scores were assessed using Pearson’s correlations (*r*). Furthermore, an exploratory stepwise multivariate linear regression analysis was performed to assess the potential contribution of each significant correlated factor (such as age, sex, MoCA, CRI, and BDI score) on OI. This stepwise method allowed us to evaluate the predictive power of each independent variable in a series of incremental models excluding the no significant ones. In the multivariate linear regression analysis, the OI scores were set as the dependent variable, while age, sex, and CRI scores were independent variables (predictors). In order to perform the multivariate linear regression analysis using a stepwise selection. In model 1, were calculated the correlation between OI score with the independent variable, age, then in model 2, were included the age and CRI. Finally, in model 3, were added the age, the CRI score, and sex. Statistical analyses performed to assess the sample size were carried out using the software GPower 3.1, while all other statistical analyses were carried out by the SPSS software version 22 for Windows (IBM, Armonk, N.Y., USA). The significance level was set at *p* < 0.05.

## Results

### Odor identification

The total sample was *n* = 633 (347 women and 286 men) with an age that ranged between 18 and 86 years. Descriptive statistics were performed to establish age-related normative values based on the OI test in Italian population (Table 1). In addition, descriptive characteristics of the sample for each Italian region are provided in Table 2. In our sample (Table 3), around 27% (*n* = 172) of participants showed an OI score lower than 12, thus indicating hyposmia [5, 43]. In detail (Table 3), the regions Tuscany and Emilia-Romagna showed a high frequency (around 50%) of olfactory deficits, whereas Sardinia, Trentino, Piedmont, and Friuli-Venezia Giulia exhibited a low frequency (around 15%) of OI impairment.

**Table 1:** Normative values for the Odor identification (OI) Sniffin’ Sticks test scores

	Total	Men	Women
Age group 18–29 years			
*N*	157	69	88
Mean	13.10	12.83	13.31
SD	1.69	1.75	1.63
Minimum	7	7	10
Maximum	16	16	16
Percentiles			
5	10	9	10
10	11	10	11
25	12	12	12
50	13	13	13
75	14	14	15
90	15	15	15.1
95	16	15	16
Age group 30–39 years			
*N*	129	58	71
Mean	12.64	12.41	12.82
SD	2.26	2.34	2.19
Minimum	3	6	3
Maximum	16	15	16
Percentiles			
5	8	7.95	8.2
10	10	8.9	11
25	12	10.75	12
50	13	13	13
75	14	14	14
90	15	15	15
95	15	15	16
Age group 40–49 years			
*N*	99	46	53
Mean	13	13.11	12.91
SD	1.95	1.79	2.10
Minimum	7	7	8
Maximum	16	16	16
Percentiles			
5	9	9	8
10	10	11	10
25	12	12	12
50	13	13	14
75	14	14	14
90	15	15	15
95	16	16	16
Age group 50–59 years			
* N*	106	47	59
Mean	12.70	12.06	13.20
SD	2.27	2.44	2.01
Minimum	6	6	6
Maximum	16	16	16
Percentiles			
5	8	6.4	8
10	9	8.8	11
25	12	11	13
50	13	12	13
75	14	14	15
90	15	15	15
95	16	15.6	16
Age group > 60 years			
*N*	142	66	76
Mean	10.82	10.74	10.88
SD	2.87	2.93	2.84
Minimum	1	1	3
Maximum	16	16	15
Percentiles			
5	5.15	5	5.85
10	7	7	7
25	9	9	9
50	11	11	11
75	13	13	13
90	14	14	14
95	15	14.65	15

*N* number, *SD* standard deviation

**Table 2:** Descriptive statistics for age in the total sample and for each Italian region

Regions	Number	% Women	Age			
			Mean	SD	Min	Max
Tuscany	94	52.1%	48.3	19.2	23	84
Sardinia	93	64.5%	40.4	17.1	20	84
Veneto	89	52.8%	42.7	15.8	18	82
Piedmont	61	57.4%	46.6	17.7	19	83
Friuli-Venezia Giulia	57	54.4%	46.8	18.1	18	86
Trentino	56	62,5%	42.1	11.2	22	64
Campania	52	50.0%	47.2	18.1	20	85
Emilia-Romagna	52	46.2%	45.8	17.4	19	79
Sicily	40	47.5%	48.2	18.3	23	82
Lazio	39	53.8%	44.3	18.3	23	81
Total sample	633	54.8%	44.9	17.3	18	86

*SD* standard deviation, *Min* minimum, *Max* maximum

**Table 3:** Percentage of olfactory deficits (OI score < 12) for each Italian region

OI score < 12			
Regions	Number	Total	% OI < 12
Total	172	633	27.2%
Tuscany	47	94	50.0%
Emilia-Romagna	29	52	55.8%
Lazio	17	39	43.6%
Veneto	17	89	19.1%
Campania	16	52	30.8%
Sardinia	13	93	14.0%
Piedmont	9	61	14.8%
Sicilia	9	40	22.5%
Trentino	8	56	14.3%
Friuli-Venezia Giulia	7	57	12.3%

### Differences between Italian regions

**Fig. 2:**
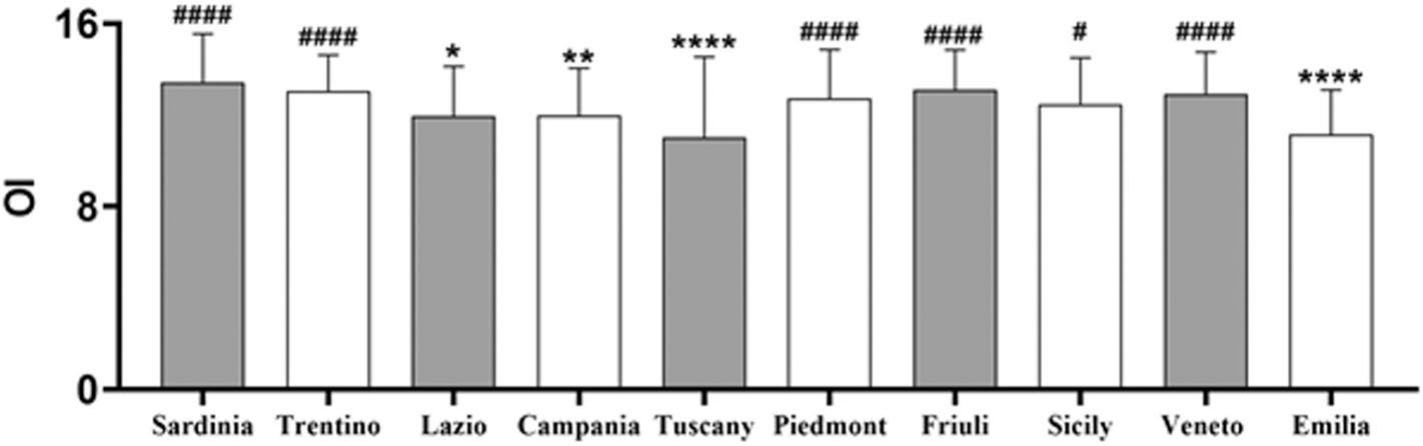
Means ± SD of olfactory identification (OI) in the ten Italian regions. One-way ANOVA followed by Tukey’s multiple pairwise comparisons. *p < 0.05; **p < 0.01; ****p < 0.0001 versus Sardinia (the region with the higher OI score); ^#^*p* < 0.05, ^####^*p* < 0.0001 versus Tuscany (the region with the lower OI score)

In Fig. 2 mean ± SD of OI score in the ten Italian regions were reported. We found a significant effect of the region factor [*F*_(9, 633)_ = 10.11, *p* < 0.0001, partial *η*^2^ = 0.127], that was still present after controlling for covariates such as sex, age, MoCA, CRI, and BDI [*F*_(9, 633)_ = 6.51, *p* < 0.0001, partial *η*^2^ = 0.087]. The Tukey’s HSD post hoc test revealed that scores found in Sardinia, the highest ones (means ± SD = 13.43 ± 2.12), were significantly higher than those in Lazio (means ± SD = 11.95 ± 2.17, *p* = 0.023, Cohen’s *d* = 0.69), Campania (means ± SD = 11.98 ± 2.07, *p* = 0.009, Cohen’s *d* = 0.69), Emilia-Romagna (means ± SD = 11.13 ± 1.97, *p* < 0.0001, Cohen’s *d* = 1.12) and Tuscany, the lower ones (means ± SD = 11.00 ± 3.53, *p* < 0.0001, Cohen’s *d* = 0.83). Similar results to Sardinia were found for Trentino (means ± SD = 13.05 ± 1.56) and Friuli-Venezia Giulia (means ± SD = 13.10 ± 1.73), with Piedmont (means ± SD = 12.72 ± 2.14), Sicily (means ± SD = 12.47 ± 2.02), and Veneto (means ± SD = 12.93 ± 1.81) displaying intermediate values.

### Effects of age and sex on Odor identification, cognitive ability, cognitive reserve index and depression

**Fig. 3:**
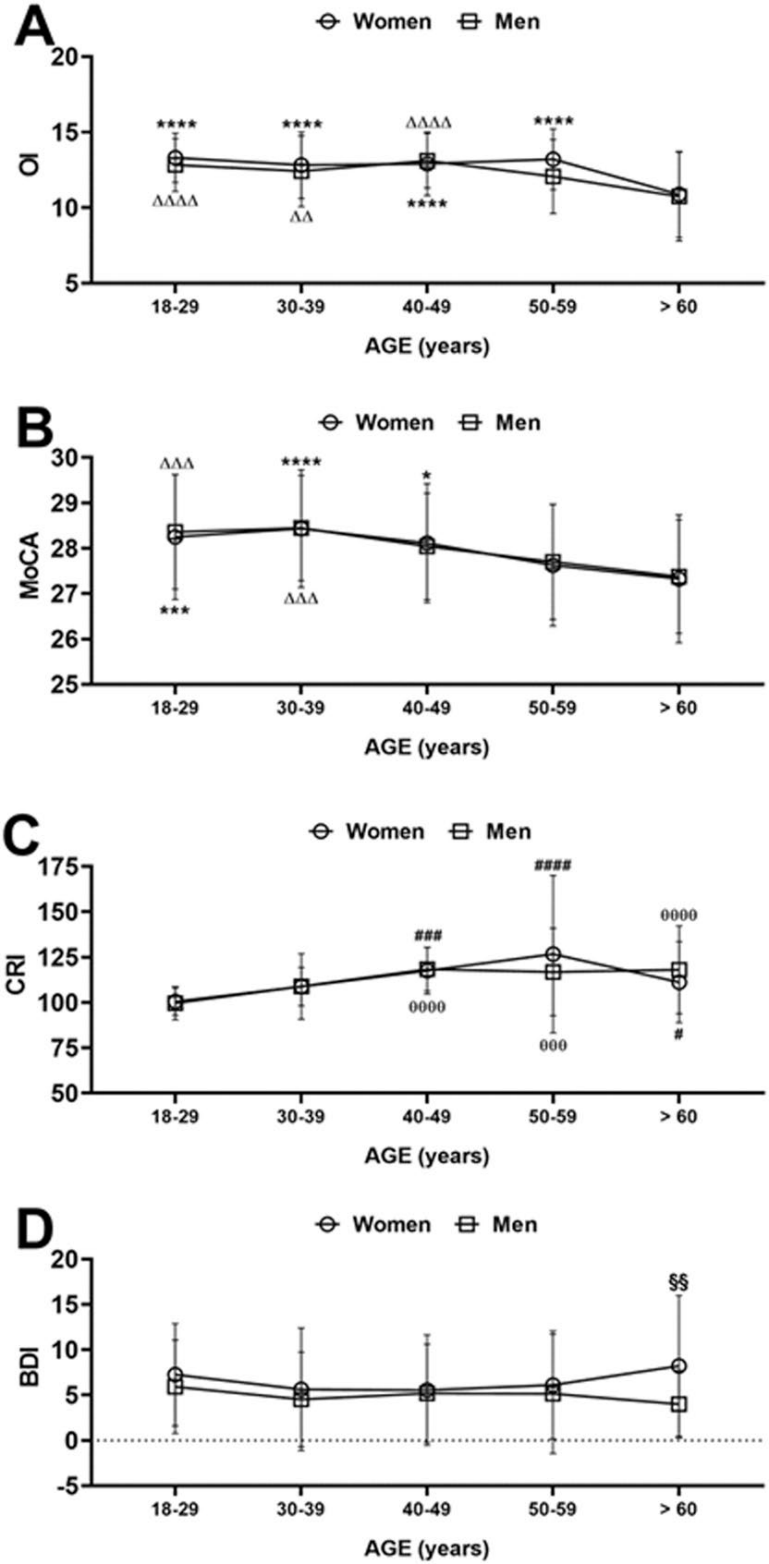
Role of sex and age on the olfactory identification (OI) (**A**), cognitive abilities (MoCA) (**B**), cognitive reserve index (CRI) (**C**) and depression level (BDI) (**D**), respectively. Data are indicated as means ± SD. Two-way ANOVA followed by Bonferroni’s or Tukey’s multiple pairwise comparisons. *p < 0.05; ***p < 0.001; ****p < 0.0001 versus age group > 60 women; ^ΔΔ^*p* < 0.01; ^ΔΔΔ^*p* < 0.001; ^ΔΔΔΔ^*p* < 0.0001 versus age group > 60 men; ^#^*p* < 0.05; ^###^*p* < 0.001; ^####^*p* < 0.0001 versus age group 18–29 women; ^θθθ^*p* < 0.001; ^θθθθ^*p* < 0.0001 versus age group > 60 men; ^§§^*p* < 0.01 women versus men

As shown in Fig. 3A, both factors, the age group and sex, had a significant effect on OI scores [age group: *F*_(4, 633)_ = 23.50, *p* < 0.0001, partial *η*^2^ = 0.131; sex: *F*_(1, 633)_ = 4.65, *p* = 0.032, partial *η*^2^ = 0.007] but not their interaction [age group × sex: *F*_(4, 633)_ = 1.29, *p* = 0.273, partial *η*^2^ = 0.008]. Bonferroni’s pairwise comparisons revealed a significant reduction of the olfactory identification in the older age group (> 60) compared to all other age groups, in both men and women (from *p* < 0.001 to *p* < 0.0001; Cohen’s *d* = 0.87 for men 18–29 vs > 60; Cohen’s *d* = 1.05 for women 18–29 vs > 60) (Fig. 3A for single points of statistical significance).

Two-way ANOVA also detected a significant effect for the age group [age group: *F*_(4, 633)_ = 16.46, *p* < 0.0001, partial *η*^2^ = 0.096], but not for the sex [sex: *F*_(1, 633)_ = 0.12, *p* = 0.733, partial *η*^2^ = 0.000] nor a significant interaction [age group × sex: *F*_(4, 633)_ = 0.83, *p* = 0.988, partial *η*^2^ = 0.000] in the MoCA test. Similarly, to data emerged for the OI, a significant age-dependent cognitive decline was observed both in men and women and along the age groups. Accordingly, in Bonferroni’s pairwise comparisons were found significant differences between the younger age groups (i.e., groups from 18–29 to 40–49) and the older ones (> 60) in both sexes (from *p* < 0.05 to *p* < 0.0001; Cohen’s *d* = 0.78 for men 18–29 vs > 60; Cohen’s *d* = 0.66 for women 18–29 vs > 60) (Fig. 3B for single statistical significances).

In the same vein, the age group [age group: *F*_(4, 633)_ = 22.23, *p* < 0.0001, partial *η*^2^ = 0.125], but not sex [sex: *F*_(1, 633)_ = 0.13, *p* = 0.714, partial *η*^2^ = 0.000], was found to influence the cognitive reserve. Nevertheless, a significant interaction effect was detected [age group × sex: *F*_(4, 633)_ = 2.51, *p* = 0.041, partial *η*^2^ = 0.016]. Accordingly, a similar trend between men and women in the age groups from 18–29 to 40–49 was found. However, in the other two age groups (i.e., 50–59 and > 60) significant sex-dependent differences were observed, in particular in men the CRI score reached a plateau at age group 50–59 staying stable in the successive > 60 age group, while it was higher in women than men in the age group 50–59, but lower in the age group > 60. Coherently, Tukey’s HSD post hoc comparisons revealed significant differences between age groups 18–29 and 40–49, 50–59, and > 60, respectively, both in women and men (*p* values from *p* < 0.05 to *p* < 0.0001; Cohen’s *d* = 1.01 for men 18–29 vs > 60; Cohen’s *d* = 0.64 for women 18–29 vs > 60) (Fig. 3C for single statistical significances).

Finally, regarding the level of depression, we found a significant effect of the sex [sex: *F*_(1, 633)_ = 10.88, *p* < 0.001, partial *η*^2^ = 0.017], but not of age [age group: *F*_(4, 633)_ = 1.39, *p* = 0.236, partial *η*^2^ = 0.009], and nor of their interaction [age group × sex: *F*_(4, 633)_ = 2.11, *p* = 0.078, partial *η*^2^ = 0.013]. Moreover, Bonferroni's pairwise comparisons showed that while in the age groups 18–29 to 50–59 men and women exhibited very similar levels of depression, a significant sex-dependent difference was observed for the age group > 60 years (*p* < 0.002, Cohen’s *d* = 0.69) (Fig. 3D for single statistical significances).

### Relation between odor identification and other variables

To evaluate the associations between OI and each other factor (age, MoCA, CRI, and BDI level), bivariate correlations were carried out (Table 4). Significant negative correlations were found between OI with age (*r* = − 0.33, *p* < 0.0001), while significant positive correlations were found with MoCA (*r* = 0.12, *p* = 0.003) and CRI (*r* = 0.21, *p* < 0.0001) scores. Conversely, no significant correlation between OI and the BDI score was observed, although the *p* value was very close to the level of significance (*p* = 0.064) (Table 4).

**Table 4:** Pearson’s correlations

Factor	Pearson’s correlation (*r*)	Significance (*p* value)
OI	1.000	–
Age	**− 0.326**	***p* < 0.01**
MoCA	**0.118**	**0.003**
CRI	**0.208**	***p* < 0.01**
BDI	0.074	0.064

Bold indicates a significant level (*p* < 0.01)

*OI* olfactory identification, *MoCA* cognitive function, *CRI* cognitive reserve, *BDI* depression level

### Multivariate linear regression analysis

Finally, to investigate the contribution of sex, age, BDI, MoCA and CRI level on the OI score an exploratory stepwise multivariate linear regression analysis was performed. The OI was considered as the dependent variable, while sex, age, MoCA, CRI, and BDI scores were used as predictors. In model 1 a significant contribution of age emerged (*F*_(1, 631)_ = 74.89, *p* < 0.0001) and the model explained around 10% of variance (*R*^2^ = 0.106). Instead, in model 2, a significant effect was observed for age and CRI (*F*_(2, 630)_ = 76.63, *p* < 0.0001) with an explanation of about 20% of variance (*R*^2^ = 0.196). Finally, in model 3 a significant contribution was observed for sex, age and CRI (*F*_(3, 629)_ = 53.22, *p* < 0.0001). Model 3 explained about 20% of variance (*R*^2^ = 0.202) (Table 5).

**Table 5:** Multiple linear regression analyses

Predictors	*B*	Std error	Beta	*t*	Significance (*p* value)
OI (dependent variable)					
Model 1					
Age	− 0.045	0.005	− 0.326	− 8.654	**< 0.0001**
Model 2					
Age	− 0.056	0.005	− 0.403	− 10.929	**< 0.0001**
CRI	0.034	0.004	0.309	8.376	**< 0.0001**
Model 3					
Age	− 0.056	0.005	− 0.403	− 10.947	**< 0.0001**
CRI	0.034	0.004	0.309	8.398	**< 0.0001**
Sex	− 0.399	0.172	− 0.082	− 2.315	**0.021**

*OI* odor identification, *CRI* cognitive reserve inventory. Bold indicates a significant level

## Discussion

Compared to the few previous studies [38–40], the present results provide new data on olfactory function in different Italian regions. Our results clearly showed significant differences between Italian regions in odor identification scores. OI scores were higher in Sardinia compared to Lazio, Campania, Emilia-Romagna, and Tuscany. The first explanation of differences in OI scores may be associated with environmental factors, pollution, agricultural and cooking practices. Moreover, olfactory function may be better trained in subjects living in natural environmental conditions. OI abilities are related to various conditions including environmental factors, previous experiences, cultural practices, or dietary behavior [11, 49, 50]. Among environmental factors, the exposure to chemical toxins and air pollution may damage the olfactory system as reported in previous studies [51–53]. In addition, temperature, humidity, altitude, and air pollution may play a role in olfactory function as indicated in a previous study [50]. The differences in odor identification abilities between Italian regions may be also associated to socioeconomic status, alcohol consumption, education level, and cognitive abilities.

The pleasantness of an odor is related to past experiences and memories [54] with familiar odors being more easily identified compared to unfamiliar ones [31, 55, 56].

Moreover, our results confirmed that OI was correlated to age, sex, and cognitive reserve index (CRI). In line to many previous studies [4, 5, 48, 57] we found a significant reduction in olfactory performance in relation to age increasing. Several theories were proposed for this age-related decrease in olfactory function, which may be related to a reduced number of olfactory receptor neurons, along with a decreased number of fibers in the olfactory bulb [28, 58], changes at a cortical level, changes in the number and in the width of the holes on the cribriform plate, or changes in the mucus composition [57]. Smell sensory loss linked to aging could impair overall health, autonomy, and quality of life as well immunity and appetite contributing also to the development of the so-called anorexia of aging [59, 60]. However, it is important to consider that an olfactory deficit in older individuals is not an inevitable fate [42]. Indeed, older people could show normal olfactory function, so the phenomenon of an age-related impairment should be better investigated, being possibly linked also to a neurodegenerative process, or side effects of drugs [61, 62].

Concerning sex differences in olfactory performance, according to many previous studies [13, 16, 43, 63], our data showed that women exhibited a better odor identification ability than men. Possible explanations for sex differences in olfactory performance were discussed in relation to the female endocrine system and estrogen effects in odor perception [14, 16]. Interestingly, no major sex-related differences were reported in the intranasal volume [64] or in the degree of expression of olfactory receptors [65].

The present results showed a positive association between olfactory function and cognitive reserve index. Previous studies [66, 67] indicated correlations between cognitive abilities and OI. In particular, Yahiaoui-Doktor and colleagues [67] showed that higher olfactory scores were associated with better verbal abilities and semantic memory. Larsson et al. [66] reported an association between age, sex, cognitive speed, and verbal abilities versus odor identification [30]. Indeed, OI task involves high-order cognitive functions and during the olfactory identification process, detection, discrimination, recognition and retrieval of an odor name are requested [30, 68]. Hence, olfactory identification score could be used as a potential early biomarker of mild cognitive impairment in clinical assessments, considering also that it is easy and quick to use without loss of time [47, 69].

The main limitation of the study is the selective use of OI, which represents only a segment of olfactory function. To obtain a more complete evaluation of olfactory performance future studies should investigate also other olfactory domains, such as odor threshold and odor discrimination.

## Conclusion

Our study provides data on the prevalence of olfactory dysfunction in a sample of Italian adults. About 27% participants showed hyposmia with an OI score < 12 and differences in OI scores were found among Italian regions. In addition, there was a clear association between OI and age, sex, and CRI with younger women with good CRI exhibiting best scores.

## References

[1] Hoskison EE (2013). Olfaction, pheromones and life. J Laryngol Otol.

[2] Croy I, Nordin S, Hummel T (2014). Olfactory disorders and quality of life—an updated review. Chem Senses.

[3] Doty RL (2009). The olfactory system and its disorders. Semin Neurol.

[4] Doty RL, Kamath V (2014). The influence of age on olfaction: a review. Front Phycol.

[5] Masala C, Saba L, Cecchini MP, Solla P, Loy F (2018). Olfactory function and age: a Sniffin’ Sticks extended test study performed in Sardinia. Chemosens Percept.

[6] Hummel T, Landis BN, Hüttenbrink KB (2011). Smell and taste disorders. GMS Curr Top Otorhinolaryngol Head Neck Surg.

[7] Santos DV, Reiter ER, DiNardo LJ, Costanzo RM (2004). Hazardous events associated with impaired olfactory function. Arch Otolaryngol Head Neck Surg.

[8] Stevenson RJ (2010). An initial evaluation of the functions of human olfaction. Chem Senses.

[9] Frasnelli J, Hummel T (2005). Olfactory dysfunction and daily life. Eur Arch Otorhinolaryngol.

[10] Hummel T, Nordin S (2005). Olfactory disorders and their consequences for quality of life. Acta Otolaryngol.

[11] Oleszkiewicz A, Kunkel F, Larsson M, Hummel T (2020). Consequences of undetected olfactory loss for human chemosensory communication and well-being. Philos Trans R Soc Lond B Biol Sci.

[12] Hummel T, Whitcroft KL, Andrews P, Altundag A, Cinghi C, Costanzo RM, Damm M, Frasnelli J, Gudziol H, Gupta N, Haehner A, Holbrook E, Hong SC, Hornung D, Hüttenbrink KB, Kamel R, Kobayashi M, Konstantinidis I, Landis BN, Leopold DA, Macchi A, Miwa T, Moesges R, Mullol J, Mueller CA, Ottaviano G, Passali GC, Philpott C, Pinto JM, Ramakrishnan VJ, Rombaux P, Roth Y, Schlosser RA, Shu B, Soler G, Stjärne P, Stuck BA, Vodicka J, Welge-Luessen A (2016). Position paper on olfactory dysfunction. Rhinology.

[13] Brand G, Millot JL (2001). Sex differences in human olfaction: between evidence and enigma. Q J Exp Psychol B.

[14] Doty RL, Cameron EL (2009). Sex differences and reproductive hormone influences on human odor perception. Physiol Behav.

[15] Larsson M, Finkel D, Pedersen NL (2000). Odor identification: influences of age, gender, cognition, and personality. J Gerontol B Psychol Sci Soc Sci.

[16] Sorokowski P, Karwowski M, Misiak M, Marczak MK, Dziekan M, Hummel T, Sorokowska A (2019). Sex differences in human olfaction: a meta-analysis. Front Psychol.

[17] Cavazzana A, Wesarg C, Schriever VA, Hummel T, Lundström JN, Parma V (2016). A cross-cultural adaptation of the Sniffin’ Sticks olfactory Identification test for US children. Chem Senses.

[18] Chrea C, Valentin D, Sulmont-Rossé C, Mai HL, Nguyen DH, Abdi H (2004). Culture and odor categorization: agreement between cultures depends upon the odors. Food Qual Prefer.

[19] Neiers F, Jarriault D, Menetrier F, Briand L, Heydel J-M (2021). The odorant metabolizing enzyme UGT2A1: immunolocalization and impact of the modulation of its activity on the olfactory response. PLoS One.

[20] Seiden AM (2004). Postviral olfactory loss. Otolaryngol Clin N Am.

[21] Masala C, Solla P, Liscia A, Defazio G, Saba L, Cannas A, Cavazzana A, Hummel T, Haehner A (2018). Correlation among olfactory function, motors' symptoms, cognitive impairment, apathy, and fatigue in patients with Parkinson's disease. J Neurol.

[22] Solla P, Masala C, Liscia A, Piras R, Ercoli T, Fadda L, Hummel T, Haenher A, Defazio G (2020). Sex-related differences in olfactory function and evaluation of possible confounding factors among patients with Parkinson's disease. J Neurol.

[23] Haehner A, Masala C, Walter S, Reichmann H, Hummel T (2019). Incidence of Parkinson's disease in a large patient cohort with idiopathic smell and taste loss. J Neurol.

[24] Stefani A, Iranzo A, SINBAR (Sleep Innsbruck Barcelona) group (2021). Alpha-synuclein seeds in olfactory mucosa of patients with isolated REM sleep behavior disorder. Brain.

[25] Croy I, Hummel T (2017). Olfaction as a marker for depression. J Neurol.

[26] Masala C, Käehling C, Fall F, Hummel T (2019). Correlation between olfactory function, trigeminal sensitivity, and nasal anatomy in healthy subjects. Eur Arch Otorhinolaryngol.

[27] Furudono Y, Sone Y, Takizawa K, Hirono J, Sato T (2009). Relationship between peripheral receptor code and perceived odor quality. Chem Senses.

[28] Buschhüter D, Smitka M, Puschmann S, Gerber JC, Witt M, Abolmaali ND, Hummel T (2008). Correlation between olfactory bulb volume and olfactory function. Neuroimage.

[29] Malnic B, Godfrey PA, Buck LB (2004). The human olfactory receptor gene family. Proc Natl Acad Sci U S A.

[30] Hedner M, Larsson M, Arnold N, Zucco GM, Hummel T (2010). Cognitive factors in odor detection, odor discrimination, and odor identification tasks. J Clin Exp Neuropsychol.

[31] Distel H, Ayabe-Kanamura S, Martınez-Gomez M, Schicker I, Kobayakawa T, Saito S, Hudson R (1999). Perception of everyday odors—correlation between intensity, familiarity and strength of hedonic judgement. Chem Senses.

[32] Konstantinidis I, Printza A, Genetzaki S, Mamali K, Kekes G, Constantinidis J (2008). Cultural adaptation of an olfactory identification test: the Greek version of Sniffin’ Sticks. Rhinology.

[33] Tekeli H, Altundağ A, Salihoğlu M, Cayönü M, Kendirli MT (2013). The applicability of the “Sniffin’ Sticks” olfactory test in a Turkish population. Med Sci Monit.

[34] Sorokowska A, Hummel T (2014). Polish version of the Sniffin’ Sticks Test—adaptation and normalization. Otolaryngol Pol.

[35] Catana I, Negoiaș S, Maniu A, Porojan M, Cosgarea M (2014). A modified version of “Sniffin’Sticks” odor identification test: the Romanian cultural adaptation. Clujul Medical.

[36] Oleszkiewicz A, Taut M, Sorokowska A, Radwan A, Kamel R, Hummel T (2015). Development of the Arabic version of the “Sniffin’Sticks” odor identification test. Eur Arch Otorhinolaryngol.

[37] Sorokowska A, Schriever VA, Gudziol V, Hummel C, Hähner A, Iannilli E, Sinding C, Aziz M, Seo HS, Negoias S (2015). Changes of olfactory abilities in relation to age: odor identification in more than 1400 people aged 4 to 80 years. Eur Arch Otorhinolaryngol.

[38] Eibenstein A, Fioretti AB, Lena C, Rosati N, Ottaviano I, Fusetti M (2005). Olfactory screening test: experience in 102 Italian subjects. Acta Otorhinolaryngol Ital.

[39] Maremmani C, Rossi G, Tambasco N, Fattori B, Pieroni A, Ramat S, Napolitano A, Vanni P, Serra P, Piersanti P, Zanetti M, Coltelli M, Orsini M, Marconi R, Purcaro C, Rossi A, Calabresi P, Meco G (2012). The validity and reliability of the Italian Olfactory Identification Test (IOIT) in healthy subjects and in Parkinson's disease patients. Parkinsonism Relat Disord.

[40] Cantone E, Ciofalo A, Vodicka J, Iacono V, Mylonakis I, Scarpa B, Russo M, Iengo M, de Vincentiis M, Martini A, Ottaviano G (2017). Pleasantness of olfactory and trigeminal stimulants in different Italian regions. Eur Arch Otorhinolaryngol.

[41] Hummel T, Sekinger B, Wolf SR, Pauli E, Kobal G (1997). ‘Sniffin’ Sticks’: olfactory performance assessed by the combined testing of odor identification, odor discrimination and olfactory threshold. Chem Senses.

[42] Hummel T, Kobal G, Gudziol H, Mackay-Sim A (2007). Normative data for the "Sniffin' Sticks" including tests of odor identification, odor discrimination, and olfactory thresholds: an upgrade based on a group of more than 3000 subjects. Eur Arch Otorhinolaryngol.

[43] Oleszkiewicz A, Schriever VA, Croy I, Hähner A, Hummel T (2019). Updated Sniffin' Sticks normative data based on an extended sample of 9139 subjects. Eur Arch Otorhinolaryngol.

[44] Nucci M, Mapelli D, Mondini S (2011). Cognitive reserve index questionnarie (CRIq): a new instrument for measuring cognitive reserve. Aging Clin Exp Res.

[45] Conti S, Bonazzi S, Laiacona M, Masina M, Coralli MV (2015). Montreal Cognitive Assessment (MoCA)-Italian version: regression based norms and equivalent scores. Neurol Sci.

[46] Beck AT, Ward CH, Mendelson M, Mock J, Erbaugh J (1961). An inventory for measuring depression. Arch Gen Psychiatry.

[47] Cecchini MP, Federico A, Zanini A, Mantovani E, Masala C, Tinazzi M, Tamburin S (2019). Olfaction and taste in Parkinson's disease: the association with mild cognitive impairment and the single cognitive domain dysfunction. J Neural Transm (Vienna).

[48] Sanna F, Loy F, Piras R, Moat A, Masala C (2021). Age-related cognitive decline and the olfactory identification deficit are associated to increased level of depression. Front Neurosci.

[49] Majid A, Speed L, Croijmans I, Arshamian A (2017). What makes a better smeller?. Perception.

[50] Guarneros M, Hudson R, López-Palacios M, Drucker-Colín R (2015). Reference values of olfactory function for Mexico City inhabitants. Arch Med Res.

[51] Mascagni P, Consonni D, Bregante G, Chiappino G, Toffoletto F (2003). Olfactory function in workers exposed to moderate airborne cadmium levels. Neurotoxicology.

[52] Yang J, Pinto JM (2016). The epidemiology of olfactory disorders. Curr Otorhinolaryngol Rep.

[53] Sorokowska A, Sorokowski P, Hummel T, Huanca T (2013). Olfaction and environment: Tsimane’ of Bolivian rainforest have lower threshold of odor detection than industrialized German people. PLoS One.

[54] Masaoka Y, Sugiyama H, Katayama A, Kashiwagi M, Homma I (2012). Slow breathing and emotions associated with odor-induced autobiographical memories. Chem Senses.

[55] Ayabe-Kanamura S, Schicker I, Laska M, Hudson R, Distel H, Kobayakawa T, Saito S (1998). Differences in perception of everyday odors: a Japanese-German cross-cultural study. Chem Senses.

[56] Hudson R (1999). From molecule to mind: the role of experience in shaping olfactory function. J Comp Physiol A.

[57] Hummel T, Oleszkiewicz A, Fritzsch B (2020). Age-related changes of chemosensory function. The senses: a comprehensive reference.

[58] Boyce JM, Shone GR (2006). Effects of ageing on smell and taste. Postgrad Med J.

[59] Schiffman S, Graham B (2000). Taste and smell perception affect appetite and immunity in the elderly. Eur J Clin Nutr.

[60] Di Francesco V, Fantin F, Omizzolo F, Residori L, Bissoli L, Bosello O, Zamboni M (2007). The anorexia of aging. Dig Dis.

[61] Nordin S, Almkvist O, Berglund B (2012). Is loss in odor sensitivity inevitable to the aging individual? A study of “successfully aged” elderly. Chemosens Percept.

[62] Mackay-Sim A, Johnston AN, Owen C, Burne TH (2006). Olfactory ability in the healthy population: reassessing presbyosmia. Chem Senses.

[63] Larsson M, Lövdén M, Nilsson LG (2003). Sex differences in recollective experience for olfactory and verbal information. Acta Physiol (Oxf).

[64] Schriever VA, Hummel T, Lundström JN, Freiherr J (2013). Size of nostril opening as a measure of intranasal volume. Physiol Behav.

[65] Verbeurgt C, Wilkin F, Tarabichi M, Gregoire F, Dumont JE, Chatelain P (2014). Profiling of olfactory receptor gene expression in whole human olfactory mucosa. PLoS One.

[66] Larsson M, Nilsson LG, Olofsson JK, Nordin S (2004). Demographic and cognitive predictors of cued odor identification: evidence from a population-based study. Chem Senses.

[67] Yahiaoui-Doktor M, Luck T, Riedel-Heller SG, Loeffler M, Wirkner K, Engel C (2019). Olfactory function is associated with cognitive performance: results from the population-based LIFE-Adult-Study. Alzheimers Res Ther.

[68] Cecchini MP, Viviani D, Sandri M, Hähner A, Hummel T, Zancanaro C (2016). Olfaction in people with Down syndrome: a comprehensive assessment across four decades of age. PLoS One.

[69] Devanand DP, Lee S, Manly J, Andrews H, Schupf N, Doty RL (2015). Olfactory deficits predict cognitive decline and Alzheimer dementia in an urban community. Neurology.

